# Successful surgical management of a through-and-through right atrial penetrating injury with a complete transaction of the right internal mammary artery: a case report

**DOI:** 10.1093/jscr/rjaa566

**Published:** 2021-01-09

**Authors:** Ibrahim Albabtain, Ali Albargawi, Sami A Almalki, Mohammed Alnasser

**Affiliations:** Department of Surgery, Ministry of the National Guard-Health Affairs, Riyadh, Saudi Arabia; King Saud bin Abdulaziz University for Health Sciences, Riyadh, Saudi Arabia; King Abdullah International Medical Research Center, Riyadh, Saudi Arabia; Department of Surgery, Ministry of the National Guard-Health Affairs, Riyadh, Saudi Arabia; King Saud bin Abdulaziz University for Health Sciences, Riyadh, Saudi Arabia; King Abdullah International Medical Research Center, Riyadh, Saudi Arabia; Department of Surgery, Ministry of the National Guard-Health Affairs, Riyadh, Saudi Arabia; King Saud bin Abdulaziz University for Health Sciences, Riyadh, Saudi Arabia; King Abdullah International Medical Research Center, Riyadh, Saudi Arabia; Department of Surgery, Ministry of the National Guard-Health Affairs, Riyadh, Saudi Arabia; King Saud bin Abdulaziz University for Health Sciences, Riyadh, Saudi Arabia; King Abdullah International Medical Research Center, Riyadh, Saudi Arabia

## Abstract

An injury to the pericardium or the great vessels is considered a true medical emergency, with a poor survival rate. The early identification and immediate response from all the medical services play a significant role in the management of this type of injury. In this case report, we report a young male patient brought to the emergency room (ER) after sustaining two stab wounds to the chest. We present the successful management of the patient from admission to the ER until discharge a few days later, after a successful surgical intervention for a penetrating cardiac injury.

## INTRODUCTION

Penetrating cardiac injuries (PCI) are considered a major surgical challenge for trauma teams due to the high mortality rate, ranging from 3% to 84% [1–3]. The high mortality rate is due to either cardiac tamponade if the pericardium is not violated or massive hemorrhage from a laceration. In a study, PCIs represented 6.4% of penetrating chest injuries [4]. Due to the anatomical position, the right ventricle is more prone to injury, as it lies more anteriorly occupying the largest anterior part of the surface of the heart. In this case report, we discuss the successful surgical management of a through-and-through cardiac injury in a young patient.

## CASE PRESENTATION

A 29-year-old man was transported by ambulance to the emergency room (ER) after receiving two stab wounds with a knife in the chest. One wound was in the left side of his chest, on the midclavicular line around four fingers superior to the nipple, and the other in the right side of his chest on the midclavicular line, two fingers inferior to the nipple. On arrival at the hospital 10 minutes after the injury, patient was awake, fully conscious and mildly tachycardiac, with blood pressure of 90/50 mmHg. There was no active external bleeding from any of the wounds.

Auscultation of the chest revealed decreased air entry on the right side. A right-sided chest tube was inserted and ~300 ml of blood drained immediately. The blood pressure dropped to 70/60 mmHg. He improved after resuscitation with 2 L of normal saline and 2 units of packed red blood cells. The right-sided chest tube drained 1300 ml of blood. A sonographic extended focused assessment for trauma revealed a right-sided hemothorax and hemopericardium.

**Figure 1:**
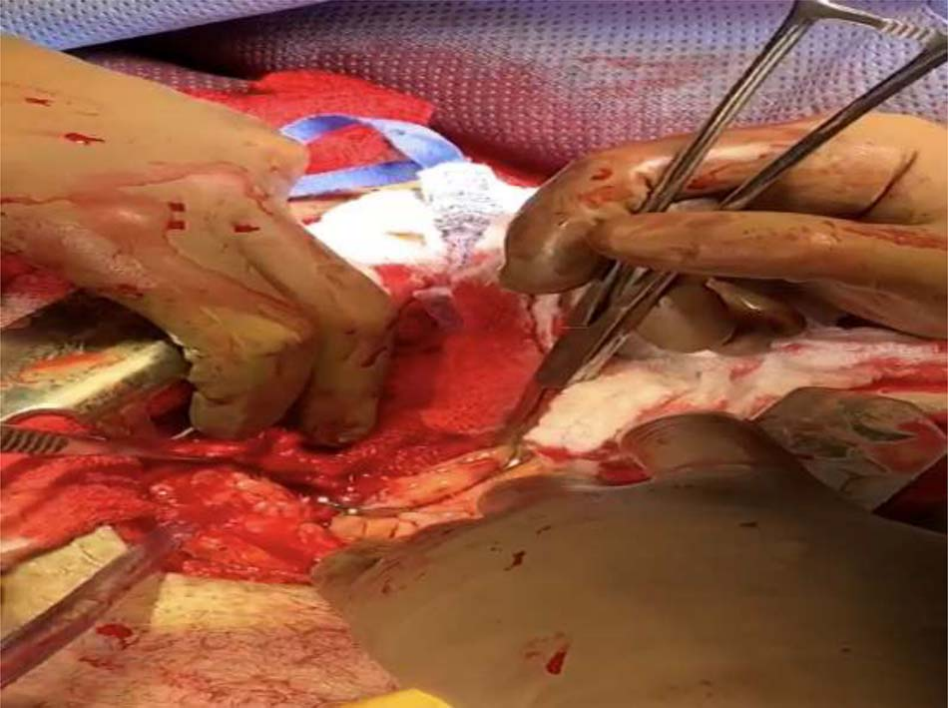
A Satinsky’s clamp is being applied over the atrial injury

The patient was transferred to the operating room. After intubation, a median sternotomy was made with an electrical saw. The right chest was packed, the pericardium opened, revealing a small through-and-through injury to the right atrium. After applying a Satinsky’s clamp, the atrial wound was closed with a pledgeted suture (Fig. 1). Bleeding was observed from a completely transacted right internal mammary artery, which was controlled with ligation. The left-sided stab wound penetrated the subcutaneous tissue, but not the pleural cavity. A diagnostic laparoscopy was done to confirm an intact diaphragm, which was difficult to rule out through a median sternotomy approach.

The patient was admitted to the intensive care unit, intubated with a minimal requirement of norepinephrine. The next day, the norepinephrine was gradually weaned off and stopped. The patient was extubated. He was transferred to a regular ward on the second postoperative day and remained hemodynamically stable with an uneventful postoperative course. The patient was discharged on the sixth day postoperatively.

Patient gave consent for the publication. All measures were taken to ensure anonymity and privacy.

## DISCUSSION

PCIs are considered a true medical emergency with an unpredictable clinical course. This case demonstrates the importance of collaboration between trauma teams and other services. Most patients with a PCI are unstable, with a poor prognosis. A study reported that 50% die before reaching the hospital [5]. For any patient presenting at the ER with a penetrating trauma to the chest, PCIs must be ruled out. In a longitudinal study with penetrating chest injuries, PCIs occurred in 6.4% [6]. The Los Angeles County + University of Southern California trauma registry from 2000 to 2010 indicates that 10% of all PCIs have an isolated right atrial injury with a survival rate of 20.5% [7].

Any injury in the cardiac box has a high risk for PCIs. The cardiac box is an area of the anterior chest delimited laterally by the midclavicular lines, superiorly by the clavicles and inferiorly by a transverse line drawn between the points where the midclavicular lines intersect the costal margins. Some patients with a cardiac injury may arrive at the ER hemodynamically stable with a deceptive clinical picture. The left ventricle may seal the myocardial injury and prevent hemorrhage due to its muscular nature. Exsanguinating hemorrhage may occur if the PCI is extensive. Approximately 90% of patients with a PCI present with signs of cardiac tamponade [8].

The choice of the incision between a median sternotomy, clamshell thoracotomy or anterolateral thoracotomy in a patient with a PCI is a challenge to most surgeons as each has its advantages and limitations. In this case, the median sternotomy incision was used because of the presence of a hemopericardium, which increases the likelihood of a cardiac injury, and the availability of a well-trained team.

## CONCLUSIONS

This case highlights the importance of collaboration between emergency physicians, nurses and the trauma teams. The immediate response, early identification and the preparedness of the operating room support an excellent outcome.

## CONFLICT OF INTEREST STATEMENT

None declared.

## FUNDING

None.

## References

[1] Kang N, Hsee L, Rizoli S, Alison P Penetrating cardiac injury: overcoming the limits set by nature. Injury 2009;40:919–27. 19442973 10.1016/j.injury.2008.12.008

[2] Naughton MJ, Brissie RM, Bessey PQ, McEachern MM, Donald JM Jr, Laws HL Demography of penetrating cardiac trauma. Ann Surg 1989;209:676–81. 2730180 10.1097/00000658-198906000-00004 PMC1494130

[3] Demetriades D, Veen BW van der Penetrating injuries of the heart: experience over two years in South Africa. J Trauma 1983;23:1034–41. 6655748

[4] Barleben A, Huerta S, Mendoza R, Patel CV Left ventricle injury with a normal pericardial window: case report and review of the literature. J Trauma 2007;63:414–6. 17693845 10.1097/01.ta.0000246954.25883.db

[5] Kaljusto ML, Skaga NO, Pillgram-Larsen J, Tonnessen T Survival predictor for penetrating 178 cardiac injury; a 10-year consecutive cohort from a Scandinavian trauma center. Scand J Trauma Resusc Emerg Med 2015;23:41. 26032760 10.1186/s13049-015-0125-z PMC4451723

[6] Mandal AK, Sanusi M Penetrating chest wounds: 24 years experience. World J Surg 2001;25:1145–9. 11571950 10.1007/BF03215862

[7] Tang AL, Inaba K, Branco BC, Oliver M, Bukur M, Salim A, et al. Postdischarge complications after penetrating cardiac injury. Arch Surg 2011;146:1061–6. 21931004 10.1001/archsurg.2011.226

[8] Grande A, Antonacci F, Aseni P Penetrating cardiac stab wounds: a case report with management algorithm and review of the literature. Emerg Care J 2018;14:63–7.

